# Influence of Particle Size on Ecotoxicity of Low-Density Polyethylene Microplastics, with and without Adsorbed Benzo-a-Pyrene, in Clam *Scrobicularia plana*

**DOI:** 10.3390/biom12010078

**Published:** 2022-01-05

**Authors:** Ana Rita Rodrigues, Nélia C. C. Mestre, Tainá Garcia da Fonseca, Paulo Z. Pedro, Camilla C. Carteny, Bettie Cormier, Steffen Keiter, Maria João Bebianno

**Affiliations:** 1 Centre for Marine and Environmental Research (CIMA), Universidade do Algarve, Campus Universitário de Gambelas, 8005-139 Faro, Portugal; arprodrigues.17@gmail.com (A.R.R.); ncmestre@ualg.pt (N.C.C.M.); tgfonseca@ualg.pt (T.G.d.F.); ppedro@ualg.pt (P.Z.P.); 2 Systemic Physiological and Ecotoxicological Research (SPHERE), Department of Biology, University of Antwerp, Groenenborgerlaan 171, 2020 Antwerp, Belgium; camilla.catarcicarteny@uantwerpen.be; 3 Man-Technology-Environment Research Centre (MTM), School of Science and Technology, Örebro University, Fakultetsgatan 1, S-701 82 Örebro, Sweden; bettie.cormier@oru.se (B.C.); steffen.keiter@oru.se (S.K.); 4 UMR CNRS 5805 EPOC, University of Bordeaux, 33405 Talence, France

**Keywords:** microplastics, benzo-a pyrene, biomarkers, IBR, health index, *Scrobicularia plana*

## Abstract

This study investigated the ecotoxicological effects of differently sized (4–6 µm and 20–25 µm) low-density polyethylene (LDPE) microplastics (MPs), with and without adsorbed benzo-a-pyrene (BaP), in clam *Scrobicularia plana*. Biomarkers of oxidative stress (superoxide dismutase—SOD; catalase—CAT), biotransformation (glutathione-S-transferases—GST), oxidative damage (lipid peroxidation—LPO) and neurotoxicity (acetylcholinesterase—AChE) were analysed in gills and digestive glands at different time intervals for a total of 14 days of exposure. In order to have a better impact perspective of these contaminants, an integrated biomarker response index (IBR) and Health Index were applied. Biomarker alterations are apparently more related to smaller sized (4–6 µm) MPs in gills and to virgin LDPE MPs in the digestive gland according to IBR results, while the digestive gland was more affected by these MPs according to the health index.

## 1. Introduction

Plastic debris are constantly found in the ocean, which come from waste mismanagement [1] or directly from fishing industries and recreational ships [2]. Plastic debris can be divided into three categories: macroplastics, microplastics and nanoplastics. Microplastics (<5 mm) (MPs) are small plastic particles used as scrubbers in cosmetics (exfoliating hand cleansers and facial scrubs), air-blasting and also secondary plastic fragments, which are derived from the breakdown of macroplastics [3,4,5].

MPs have been recognized as ubiquitous contaminants in marine ecosystems and scientific evidence has revealed their urgent threat relative to animal and human health [6,7,8,9].

In addition to physical stress afforded by the ingestion of MPs resulting in laceration, inflammation processes and starvation, impacts are also devoted to the intake of additive or sorbed hazardous chemical contaminants adsorbed onto MPs’ surface, thus becoming a carrier for transporting and releasing substances in marine organisms [10,11,12,13].

Hydrophobic organic contaminants have a greater affinity for plastics of different chemical composition, such as polyethylene, polypropylene and PVC, than for natural sediments [14]. MPs collected on beaches worldwide have been revealed to be associated to a large amount of organic contaminants, which are known to desorb from its surface towards the water column and to be transferred over the food chain, posing additional risks to marine biota [15]. Benzo-a-pyrene (BaP) is the most commonly studied polycyclic aromatic hydrocarbon (PAHs) listed as persistent toxic substances by United Nations Environment Program and by the European Union. BaP is ubiquitously distributed in the marine environment and, due to its lipophilic nature, it is prone to bioaccumulate [16]. It represents a human genotoxic carcinogen with tumorigenic potential for organisms under exposure to such chemical [17].

In order to clarify the effects of exposure of organic contaminants, such as BaP, adsorbed to MPs on marine organisms, a set of biomarkers have been used, which includes the following: the activity of the enzymes superoxide dismutase (SOD), catalase (CAT) acting as a defense mechanism against reactive oxygen species (ROS) formation and glutathione-S-transferases (GST) mainly involved in cell detoxification; all of this work to avoid oxidative stress. Lipid peroxidation (LPO) is the process in which free radicals react in cell membranes and form lipid hydroperoxides resulting in cell damage, when oxidative stress reaches a certain level that may cause oxidative damage to DNA, proteins, carbohydrates and lipid membranes.

Acetylcholinesterase (AChE) is an essential enzyme in the transmission of nerve impulses involved in the catalytic breakdown of acetylcholine and other choline esters; thus, they are frequently applied to assess neurotoxic effects triggered by exposure to contaminants [18,19].

*Scrobicularia plana* is a suspension-feeder that not only primarily feeds on particles of surface deposits but also obtains part of the food by filtering suspended matter from the overlying water column [20]. This species is a good indicator of bioaccumulation due to their high tolerance to chemical exposure and sedentary lifestyle [21] and is an environmentally relevant species for evaluating the health of coastal environmental systems; for this reason, it was selected for assessing MP responses.

The aim of this study was to determine whether biological impacts provoked by exposure of low-density polyethylene (LDPE) MPs to clam *S. plana* relies on the size of plastic particles, the presence of BaP adsorbed onto MPs or the combination of both. Ecotoxicological effects were investigated in *S. plana* when exposed to contaminated and uncontaminated LDPE MPs of two different sizes of MPs (4–6 µm and 20–25 µm), with and without BaP adsorbed for 14 days. For that, a set of biomarkers responses was analysed, including quantification of oxidative stress (SOD and CAT activities); biotransformation (GST activity); oxidative damage (LPO); and neurotoxicity (AChE activity), either in clam gills or digestive glands. The overall health status of the organism was assessed by using the condition index. In order to have a better impact perspective for the effect of both these contaminants in this bivalve species, an integrated biomarker response index (IBR) [22,23] and Health Index (HIS) were applied [24].

## 2. Materials and Methods

### 2.1. Preparation and Characterization of Microplastics

Microplastics of sizes 4–6 µm (MPP-635G) and 20–25 µm (MPP-1241) were obtained from MicroPowders Inc. (Tarrytown, NY, USA). Benzo[a]pyrene, CAS 50-32-8 (purity ≥ 96%), was purchased from Sigma Aldrich and used at a concentration of 2500 μg L^−1^. Sorption of BaP onto microplastic particles was conducted by the Man-Technology-Environment Research Centre, Department of Natural Science, Örebro University, Sweden, as described by O’Donovan et al. [9]. Briefly, 4 g of microplastics was spiked with BaP, using 125 g L^−1^ of plastic, weighted into separate 250 mL narrow mouth Septa bottles (Thermo Scientific, Waltham, MA, USA), and double deionized water was added to obtain 125 g L^−1^. Subsequently, the solution was spiked with 80 μg of BaP for the adsorption experiment. Bottles were placed on a rotary shaker for two days at the lowest speed of 20 rpm. Samples were filtered with a ceramic funnel and glass microfiber filters (1.0 μm, Whatman^®^ glass microfiber filters, GE Healthcare Life Sciences, Maidstone, UK) and rinsed afterwards with double-deionized water, dried by vacuum evaporation, and extracted in hexane (≥98%, SupraSolv, Munich, Germany). Extracts were sonicated, centrifuged at 2000× *g* RCF, filtrated through fiberglass and transferred to toluene (purity 96%, SOLVECO). Sample volume was reduced to 500 μL using liquid nitrogen, and BaP concentration was quantified using a high-resolution GC-MS system (Micromass Autopspec Ultima, Milford, MA, USA), separation on a 30 m (0.25 mm i.d., 25 μm film thickness) DB-5MS column (J&W Scientific, Folsom, CA, USA). Details about the instrumental method can be found in Larsson et al. [25]. Benzo[a]pyrene-d12 in toluene was used as an internal standard, and BaP concentration was quantified against perylene-d12 recovery standard, dissolved in toluene and purchased from Chiron. The final concentration of BaP adsorbed to LDPE (4–6 and 20–25 μm) was 16.64 ± 87 (±0.08 and 15.07 ± 0.24 μg g^−1^, respectively, to mimic similar concentrations found in the environment.

### 2.2. Experimental Design

Clams *S. plana* were collected during low tide in Cabanas de Tavira at the Ribeira do Almargem, in the Ria Formosa lagoon, (N 37°7′59.75″ W 7°36′34.95″) and immediately transported alive to the laboratory. Clams (*n* = 420) were collected in April, when there was no sexual maturity of individuals. Before sampling clams, the top 30 cm of the sediments was collected from the same site and passed through a sieve (4 mm) to eliminate possible macro-organisms and sediment debris. Afterwards, the sediment was dried at 65 °C for 48 h and re-hydrated afterwards using the percentage of water present when collected [26].

Twenty-five L aquaria each were filled with 4 L of pre-treated sediments and 16 L of natural seawater from the Ria Formosa Lagoon. Constant aeration was supplied using glass pipettes at the end of the aeration tubes in order to avoid plastic contact with the seawater. In the laboratory, air temperature was maintained at 19 °C. Clams were acclimatized over 5 days, with a photoperiod of 12 h light to 12 h dark.

The exposure experiment was conducted in a duplicate design. The aquaria included the following: control (consisting of only seawater), virgin LDPE MPs of sizes 4–6 μm and 20–25 μm, BaP-contaminated LDPE MPs of sizes 4–6 μm and 20–25 μm at a concentration of MPs of 1 mg L^−1^. The bioassay lasted only 14 days, and samples were collected at different times of exposure: at the beginning of the experiment and at the 7th and 14th days. During the bioassay, seawater was renewed every 48 h, with reintroduction of the same concentration of respective MPs types for each treatment. No food was given during the exposure in order to minimize interaction with organisms and microplastics. Temperature (19.3 ± 0.2 °C), salinity (35 ± 1), percentage of oxygen saturation (95.8 ± 1.8%) and pH (8.05 ± 0.04), were measured every two days by using a multiparametric probe (ODEON V3.3.0).

At each sampling time, clams *S. plana* were randomly collected from each aquarium before water change and MP addition. On day 0, before the addition of microplastics (MPs), 30 clams per treatment were collected, with no distinction being made between aquarium for the determination of SOD, CAT, GST and AChE activities; LPO; condition index (CI); BaP; and MP accumulation. After 7 days of exposure, clams were collected from each aquarium for the analysis of SOD, CAT, GST and AChE activities; LPO; and CI.

At the 14th day of exposure, clams were collected for analysis of the same biomarkers and for BaP and MPs accumulation. At each sampling time, gills and digestive gland were dissected immediately, flash frozen in liquid nitrogen and stored at −80 °C for further analyses. For MP quantification and BaP analysis, the entire soft tissue was stored in aluminum foil and kept at −20 °C.

### 2.3. Condition Index

Condition index (CI) was measured in 6 individuals from each treatment and time of exposure. At day 0 (pre-exposure) and after 7th and 14th day of exposure, 6 individuals were randomly sampled from each aquaria to assess the physiological status of clams. Tissues were dried in an oven at 75 °C until constant dry weight was achieved. CI was calculated as the percentage (%) of the ratio between dry weight of the soft tissues (g) and the dry weight (g) of the shell [27].

### 2.4. MPs Quantification

MPs quantification in the whole soft tissues (whole animal minus shell) of *S. plana*, was assessed on 6 clams collected at the beginning of the experiment and on the same number of clams collected after the 14th day of exposure and stored in aluminum foil and kept at −20 °C until further analysis. Samples were dried for 48 h at 75 °C to obtain dry weight (g). In a laminar flow hood, samples were placed, individually, in glass flasks. An amount of 3 mL of nitric acid (69%) was used to digest organic matter for 24 h. An extra 5 mL of nitric acid was added, and flasks were placed in a heating plate, at 60 °C, to evaporate nitric acid as much as possible without losing sample material. During the entire process, each flask was covered with aluminum foil to avoid contamination.

After digestion, 20 mL of pre-filtered distilled water was added in each flask to dilute nitric acid, preventing filter damage. In order to eliminate unwanted particles, density separation was performed using a NaCl solution (140 M). Each sample was kept in this solution for 1 h in a beaker, along with eight drops of a Nile Red dye (Sigma Aldrich, CAS 553-24-2, St. Louis, MO, USA) solution 0.4% in PBS. Nile Red is a lipophilic stain that was used to bind to MPs. MPs dyed with Nile Red when exposed to ultraviolet light emit fluorescence, making the counting of MPs possible and easier. Each sample, after being separated by density, was filtered using 0.2 µm cellulose filters to prevent MPs loss. Filtration was performed in a laminar flow hood and only glass material was used during the entire procedure. MPs quantification was assessed in a fluorescence microscope, Leica DMLB. Each filter had 100 grids, but due to the number of MPs in each filter, only 30 grids were counted, and an estimation was made afterwards for the 100 grids.

### 2.5. Quantification of BaP Accumulation

In order to quantify BaP accumulation in entire soft tissues of freeze-dried clams, the method previously described by De Witte et al. [28] was used. Six clams per treatment collected at the beginning and end of the experiment (days 0 and 14) were analysed. Samples were extracted by accelerated solvent extraction (Dionex, ASE350, Sunnyvale, CA, USA), using a mixture of hexane and acetone 3:1, at 100 °C. After two evaporation steps, 5 μL of each sample was injected on a GC (Agilent 7890A) equipped with a PTV-injector (Gertsel, 6495-U, Sursee, Switzerland). An Agilent 5975C MS-detector with electron impact ionization in single-ion mode was used for detection. As an internal standard, chrysene-d12 in toluene was added to the vial. Quantification was performed against benzo[a]pyrene-d12 recovery standard, dissolved in iso-octane and purchased from LGC-standards. The analysis is accredited by BELAC under the ISO/IEC 17025 standard, with a quantification limit of 1.65 ng g^−1^ d.w. [9].

### 2.6. Enzyme Activity

#### 2.6.1. Homogenization of Tissues

For the determination of enzyme activities, gills and digestive glands of six individual clams previously collected from each treatment and time of exposure (day 0 (pre-exposure) and 7th and 14th days of exposure) were used. Gills and digestive glands previously stored at −80 °C were thawed on ice, weighted and homogenized on ice in 5 mL of Tris sucrose buffer (Sucrose 0.5 M, Tris 20 mM, KCL 0.5 M, DTT 1 M, EDTA 1 mM, at pH 7.6). The homogenate was centrifuged at 500× *g*, for 15 min at 4 °C. The supernatant was transferred to another centrifuge tube and centrifuged a second time at 12,000× *g*, for 45 min at 4 °C. Supernatant was recovered and divided into four aliquots and stored in microcentrifuge tubes for posterior analysis of SOD, CAT and GST activities and total protein concentration.

#### Superoxide Dismutase (SOD) Activity

SOD activity was determined by using the colorimetric method described by McCord and Fridovich [29]. An amount of 2.65 μL of phosphate buffer (50 mM, with EDTA 0.1 mM, at pH 7.8), 100 μL hypoxanthine (1.5 mM), 100 μL of cytochrome *c* oxidase (0.15 mM) was added to 50 μL of the homogenate of each tissue, as well as 100 μL of xanthine oxidase (56 mU/mL), and vortexed. The percentage of inhibition of the absorbance of cytochrome *c* as a result of the superoxide anion generated by the xanthine/hypoxanthine reaction was measured at 550 nm for 1 min using a spectrophotometer. Samples were run in triplicate. SOD activity is expressed in Units (U) mg^−1^ protein, where 1 U of activity equals to the quantity of sample required to cause 50% inhibition.

#### Catalase (CAT) Activity

CAT activity was determined by measuring changes in absorbance at 240 nm that corresponds to the consumption of hydrogen peroxide (H_2_O_2_), according to Greenwald [30]. For 100 μL of the homogenate of gills and digestive gland collected at each time of exposure and prepared as described in Section 2.6.1, 1900 μL of phosphate buffer (pH 7.5) and 1000 μL H_2_O_2_ were added and vortexed. The absorbance was read in a spectrophotometer at 240 nm in two replicates per tissue sample. CAT activity is expressed in μmol min^−1^ mg^−1^ of total protein concentration.

#### Glutathione-S-Transferase (GST) Activity

GST activity was determined on the cytosolic fraction of the clam gills and digestive gland following method described by Habig et al. [31], adapted to the microplate reader by McFarland et al. [32]. Glutathione-S-transferases catalyze nucleophilic attack by reduced glutathione (GSH) on nonpolar compounds that contain an electrophilic carbon, nitrogen or sulphur atom [33]. All GST families contain members that catalyze the conjugation of 1-chloro 2,4 dinitrobenzene (CDNB) and exhibit glutathione peroxidase activity toward hydroperoxide (CuOOH). Dinitrophenyl thioether is produced as a result of the reaction, for which its absorbance was detected at 340 nm in a microplate reader Tecan (Infinite 200 Pro). Samples were run in triplicate, and absorbance was read every 30 s over a 3-min period at room temperature. GST activity is expressed in nmol CDNB min^−1^ mg protein^−1^.

### 2.7. Acetylcholinesterase (AChE) Activity

The determination of AChE activity was assessed individually in the gills of six clams from each treatment collected at day 0 (pre-exposure) and after the 14th day of exposure (six replicates per treatment) according to Ellman’s colorimetric modified protocol [34], where thiocholine is produced as AChE hydrolyses to acetylcholine. The gills were defrosted on ice, weighted and homogenized in 5 mL of Tris HCl buffer (100 mM, pH 8.0) and 50 μL of Triton-X 100 (0.1%). The resulting homogenate was centrifuged at 12,000× *g* for 30 min at 4 °C. After centrifugation, the supernatant was divided into aliquots and placed in two microcentrifuge tubes and stored at −80 °C. The first aliquot was used for the determination of AChE activity and the second for the determination of total protein concentration. In order to determine AChE activity, 50 μL of each gill homogenate was introduced, in triplicate, to each well of a 96-well microplate together with 200 μL of 5,5′-dithio-bis (2- nitrobenzoic acid) (DNTB, 0.75 mM) and incubated for 5 min at room temperature. Then, 50 μL of acetylthiocholine solution (3 mM) was added to each well and incubated for 10 min at room temperature to trigger a reaction. Thiocholine reacts with DNTB, releasing 5-mercapto-2-nitrobenzoate compound for which its color is yellow. The increase in absorbance of this yellow compound was measured at 405 nm using a microplate reader Tecan (Infinite 200 Pro) in 30 s intervals for 5 min, and an extinction co-efficient of ε = 13.6 mM ^−1^ cm^−1^ was used to estimate the quantity of thiocholine produced, which is proportional to AChE activity [35]. AChE activity is expressed in nmol ACTC (acetylthiocholin) min^−1^ mg protein^−1^.

### 2.8. Lipid Peroxidation (LPO)

LPO was assessed in the gills and digestive gland of six individuals collected from each treatment at day 0 (pre-exposure) and after the 7th and 14th day of exposure (six replicates per treatment), according to the colorimetric method reported by Erdelmeier et al. [36]). Both tissues were defrosted on ice, weighted and homogenized with 5 mL of Tris HCl buffer (0-02 M, pH 8,6) and 50 μL of butylated hydroxytoluene solution (BHT). The homogenate was centrifuged at 30,000× *g* for 45 min at 4 °C. The supernatant was divided into two aliquots that were stored at −80 °C. The first aliquot was used for the determination of LPO levels and the second for the quantification of total protein concentration.

In order to determine LPO levels, 200 μL of gills and digestive gland homogenate from control, those exposed to LDPE 4–6 and 20–25 μm size range virgin MPs and those adsorbed to BaP MPs of the same size were mixed with both 650 μL of 1-methyl-2-phenylindone diluted in methanol and 150 μL of methanesulfonic acid (15.4 M) and placed into a water bath at 45 °C for 60 min. The mixture was centrifuged at 15,000× *g* for 10 min at 4 °C. An amount of 150 μL of the supernatant was added in quadruplicate to a 96-well microplate. The following reaction was used to determine lipid peroxidation by quantifying malondialdehyde (MDA) and 4-hydroxyalkenals (4-HNE) absorbance, resulting from the decomposition by polyunsaturated fatty acid peroxides: two moles of N-methyl-2-phenylindole (chromogenic reagent) and one mole of MDA were added and incubated at 45 °C for 60 min. Malondialdehyde bis (dimethyl acetal) was used as a standard. The absorbance was read using Tecan (Infinite 200 Pro) at 386 nm in a microplate reader. LPO is expressed as nmol mg protein^−1^.

### 2.9. Total Protein Concentration

In order to determine the total protein concentration in cytosolic fraction of the gills and digestive gland, the Bradford method was used [37]. The principle of the method is based on the absorbance shift of Coomassie Brilliant Blue G-250 dye. Standard protein dilutions were prepared, from 0.005–1.0 mg mL^−1^, using bovine serum albumin (BSA) for calibration. Milli-Q water was used as a blank. The total protein concentration in the gills and digestive gland defrosted on ice was diluted 1/5 with Milli-Q water and vortexed. An amount of 5 μL of each sample tissue, blank or standard was added, in quintuplet, to a 96-well microplate reader. An amount of 200 μL of diluted Bradford solution (1:5) was added to each well. The microplate was incubated in a microplate reader using Tecan (Infinite 200 Pro) and absorbance was determined at 595 nm for 20 min at room temperature. The increase in absorbance is proportional to the amount of bound dye and, therefore, to the amount of protein present in the sample. Total protein concentrations are expressed as mg g^−1^ of wet tissue weight.

### 2.10. Integrated Biomarker Response Index (IBR)

Integrated biomarker response index relies on assessing biological effects measured with a set of biomarkers and CI from gills and digestive gland tissues. This method was first defined by Beliaeff and Burgeot [22]), described by Serafim et al. [23] and later modified by Sanchez et al. [38], which is now called IBR version 2. A sum of the deviations between the different treatments with different LDPE MPs sizes with and without BaP from each individual biomarkers and CI (X_i_) was compared to the data of each biomarker and CI of the control group (X_0_) and log transformed (Y_i_) to reduce variance (Y_i_ = log (X_i_/X_0_)). Mean (μ) and standard deviation (σ) were calculated for Y_i_ and data standardized using the following formula (Equation (1)).

Z_i_ = (Y_i_ − μ)/σ (1)

Afterwards, the next area (A_i_) was calculated as the difference between the mean of standardized biomarker response and CI (Z_i_) of each exposed group and the mean of the unexposed biomarker data and CI (Z_0_) (Equation (2)).

A_i_ = Z_i_ − Z_0_ (2)

Lastly, in order to obtain the IBRv2 index, the absolute value of A_i_ summed for each parameter in each experimental condition is describe in Equation (3).

IBR = Σ|A_i_| (3)

### 2.11. Health Index (HIS)

The Health Index (HIS) was developed and calibrated using biomarkers from active biomonitoring programs and subsequently applied on datasets obtained from mesocosms experiments or field studies. The aim of the present method is to develop and test an objective decision-support expert system capable of integrating biomarker results into a five-level health-status index. The expert system is based on a set of rules derived from available data on responses to natural and contaminant-induced stress of marine animals. Integration of parameters includes the level of biological organization; biological significance; mutual inter-relationship; and qualitative trends in a stress gradient. Details of the expert system and algorithm description are in Dagnino et al. [24]. The health index was applied in the gills and digestive gland considering biomarkers over a stress gradient yield characteristic trend such as the following: bell-shaped (SOD, CAT and GPx), increasing (LPO) or decreasing (AChE and CI). The results were integrated into a five-level health status index such as A (high), B (good), C (moderate), D (poor) and E(bad), following the classification established by the European Union Water Framework Directive.

### 2.12. Statistical Analysis

Data are expressed as mean ± standard deviation (SD). Statistical differences between treatments (control, 4–6 μm MPs, 4–6 μm MPs + BaP, 20–25 μm MPs and 20–25 μm MPs + BaP), time of exposure and different tissues (gills and digestive gland) were assessed for normal distribution by homogeneity by using the Shapiro–Wilk and Kolmogorov–Smirnov parametric tests. After two-way ANOVA was used. Significant ANOVA results were analyzed using Tukey’s HSD test. Principal components analysis (PCA) was performed using PAST 3. All tests were performed using Graphpad Prism 8. Any difference with a *p* value ≤ 0.05 was considered significant.

## 3. Results

### 3.1. Condition Index

The CI of the organisms showed no significant differences between treatments for the same exposure day (*p* > 0.05) (Table 1). Results indicate that during this exposure period, no major changes in tissue or shell weights occurred that could result in a significant shift in CI.

### 3.2. MPs Quantification

**Figure 1:**
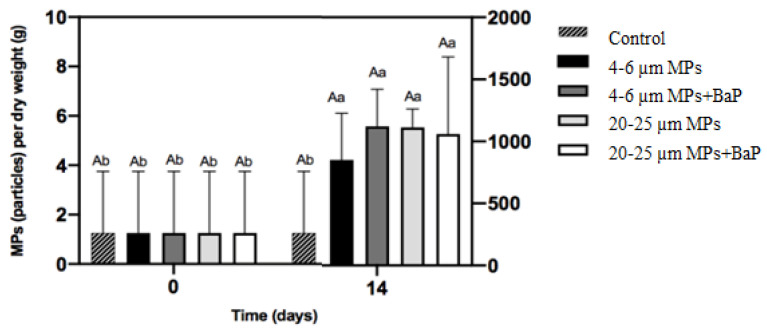
MPs accumulation per gram of dry weight (mean ± SD) of the soft tissues of *S. plana* after 14 days of exposure. Different capital letters indicate a significant difference between treatments within the same time. Different lowercase letters indicate a significant difference for the same treatment between times (*p* < 0.05).

MPs ingested in clams exposed to different sizes of MPs with and without BaP are in Figure 1. MPs ingested in MP-exposed clams showed no significant differences between treatments after 14 days (*p* < 0.05).

### 3.3. Benzo-a Pyrene Accumulation

BaP accumulation in clams’ whole soft tissues from different treatments are in Table 2. Data show that BaP levels in controls and virgin MPs were of the same order of magnitude. In clams exposed to MPs contaminated with BaP, BaP accumulation was higher than in controls and virgin MPs indicating that BaP was accumulated in clam tissues. BaP concentration was also higher in clams exposed to 20–25 μm MPs than in the lower size class.

### 3.4. Enzyme Activity

#### 3.4.1. Antioxidant (SOD and CAT) Enzymes Activity

**Figure 2:**
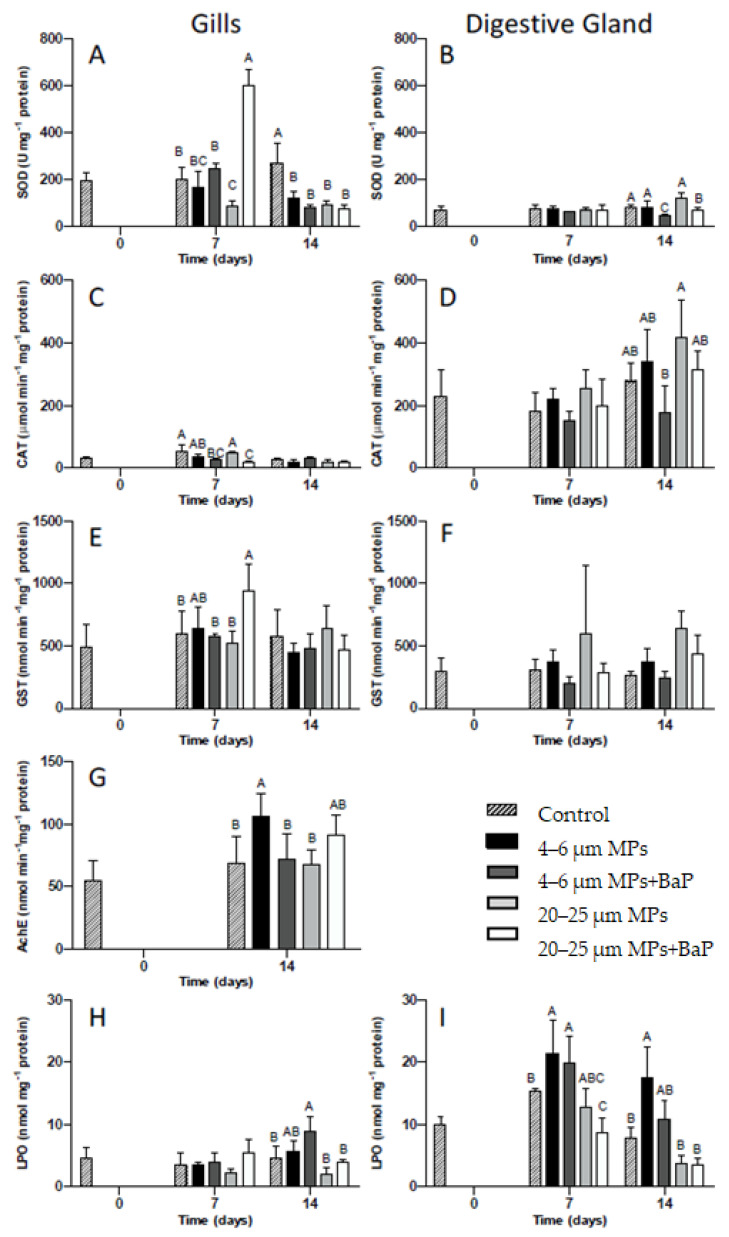
SOD (**A**), CAT (**C**), GST (**E**), AChE (**G**) activities and LPO (**H**) levels (mean ± SD) in the gills and SOD (**B**), CAT (**D**), GST (**F**), and LPO (**I**) levels in the digestive gland of *S. plana* unexposed and exposed to 4–6 µm and 20–25 µm MPs with and without BaP after 14 days of exposure. Different capital letters indicate a significant difference between treatments within the same time. (*p* < 0.05).

SOD and CAT activities in clam gills and digestive gland for each MPs treatment with and without BaP (control, 4–6 μm MPs, 4–6 μm MPs + BaP, 20–25 μm MPs and 20–25 μm MPS + BaP) at different times are displayed in Figure 2. On day 7, clams exposed to 20–25 μm MPs + BaP have higher SOD activity when compared to other treatments (*p* < 0.05). In clams exposed to 20–25 μm MP treatment, there is a significant decrease in SOD activity on day 7 when compared to other treatments, except for clams exposed to 4–6 μm MPs on the same day (*p* < 0.05). On day 14, all MP treatments have lower SOD activity compared to controls (*p* < 0.05, Figure 2A).

In relation to the digestive gland (Figure 2B), SOD activity is lower than in the gills. SOD activity in the digestive gland significantly decreases in clams exposed to 4–6 μm MPs + BaP and 20–25 μm MPs + BaP at the 14th day of exposure when compared to the other treatments (*p* < 0.05).

CAT activity is much higher in the digestive gland than in the gills (Figure 2C,D). CAT activity in the gills, only shows a significant decrease for clams exposed to 4–6 μm MPs + BaP and to 20–25 μm MPs + BaP at the 7th day (*p* < 0.05) when compared to controls (Figure 2C). In the digestive gland, only in clams exposed to 4–6 μm MPs + BaP after 14 days of CAT activity was it significantly lower than 20–25 μm MPs (*p* < 0.05) (Figure 2D).

#### 3.4.2. GST Activity

On day 7, a significant increase in GST activity was noticed in the gills of the clams exposed to 20–25 μm MPs + BaP, when compared to the other treatments (*p* < 0.05) (Figure 2E). GST activity for the digestive gland had no significant differences between treatments (*p* > 0.05) (Figure 2F).

### 3.5. Acetylcholinesterase (AChE) Activity

After 14th day of exposure there is significantly higher AChE activity in clam gills exposed to 4–6 μm MPs when compared to controls and to 20–25 μm treatments (*p* < 0.05) (Figure 2G).

### 3.6. Lipid Peroxidation (LPO)

Only a significant increase in LPO levels occurred in the gills of *S. plana* exposed to 4–6 μm MPs + BaP at day 14th (*p* < 0.05), when compared to the other treatments (Figure 2H). In the digestive gland of individuals contaminated with 4–6 μm MPs, LPO levels were significantly higher at day 14 (*p* < 0.05), when compared to the other treatments except 4–6 μm MPs + BaP. Similarly, LPO levels were significantly higher from controls (Figure 2I).

### 3.7. PCA

**Figure 3:**
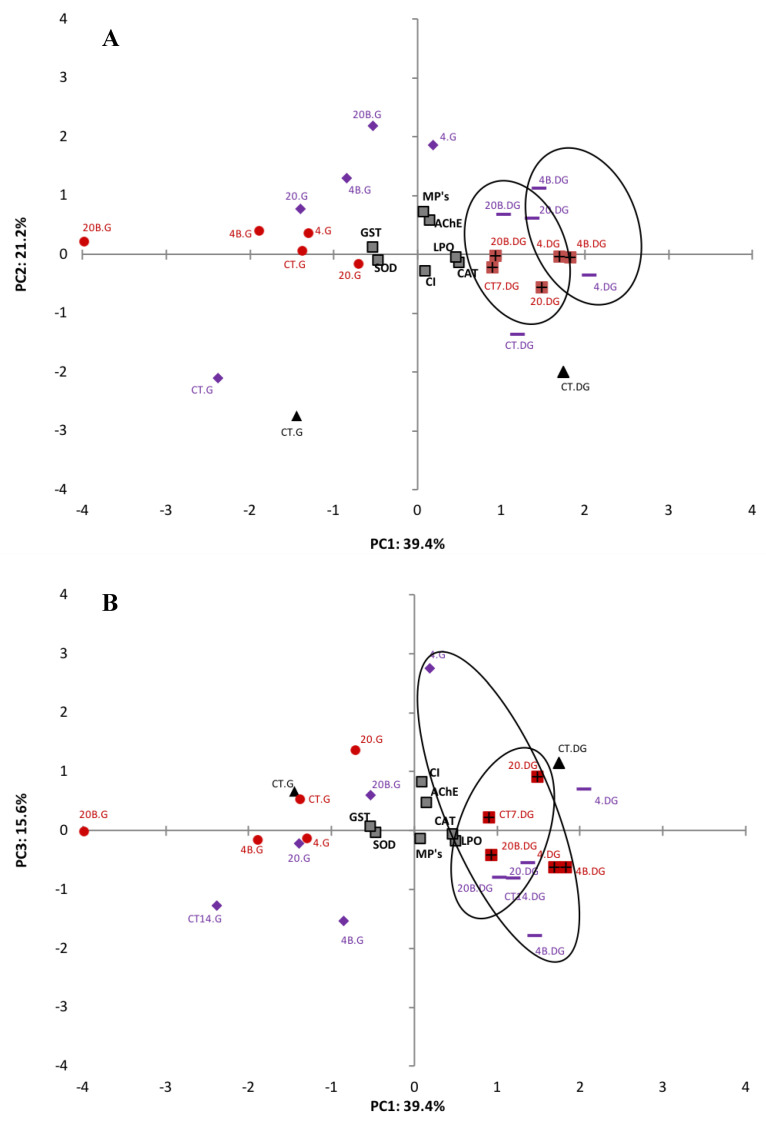
PCA of biomarkers (SOD, CAT, LPO, AChE and GST) in the gills (G) and digestive gland (GD) of the clam *S. plana*, exposed with different sizes of LDPE MPs with (4–6 μm (4) and 20–25 μm (20)) and without BaP adsorbed at different times of exposure (T0 (black), T7 (red) and T14 (purple)) (**A**) PC1 and PC2 components, (**B**) PC1 and PC3 components (*p* < 0.05).

Exploratory statistical analyses, namely PCA, were performed on all data obtained for each tissue analysed to help elucidate the effects of microplastics on the biomarkers investigated (Figure 3). To have a more confident variance, the percentages of three factors were used, which represent 76.2% of total variance. Figure 3A represents the relation between PC1 (39.4%) and PC2 (21.2%) and Figure 3B between PC1 (39.4%) and PC3 (15.6%). Regarding PC1, MPs, LPO levels, AChE activity, CAT activity and CI are positively related to digestive gland treatments, while for the gills this relationship was negative. SOD and GST activities are in the negative side of PC1 and have a negative relationship with the digestive gland of the different treatments and a positive relationship with gills treatments. All data for the digestive gland remain on the positive side of PC1 where there is a clear separation of different sizes (4–6 μm and 20–25 μm). Gills data are on the negative side with no clear size grouping. MPs, AChE activity, LPO and GST activity are in the positive side of PC2 and are positively related to the majority of the treatments. However, SOD activity, CAT activity and CI are in the negative side of PC2 and negatively related mainly to the control treatments. In relation to PC3 (Figure 3B), only MPs and CAT activity had a negative correlation, when compared to the other biomarkers. There is no clear separation between time, size of LDPE MPs or between contaminated/uncontaminated clams.

### 3.8. IBR

IBR was calculated using all biomarkers and CI data for the gills and digestive gland of clams exposed to LDPE MPs with and without adsorbed BaP, and data are included on Table 3. The IBR index was higher in the gills of clams exposed to 4–6 μm LDPE MPs in both contaminated with BaP or not, when compared with those of the bigger size. In the digestive gland, however, there was no size effect and IBR was lower in BaP-contaminated LDPE MPs when compared with uncontaminated MPs.

### 3.9. Health Index

The health index was applied in the gills and digestive gland using the same variables as in the case of IBR. HIS results (Table 3) indicate that the gills of clams exposed to 4–6 μm MPs with BaP adsorbed were considered in a pathological state while the other treatments gills were considered healthy. In the digestive gland, the HIS index was different; clams exposed to LPDE of 4–6 μm were considered in a pathological state, while those 4–6 μm MPs + BaP and 20–25 μm MPs were considered to be in medium stress, and in clams exposed to 20–25 μm MPs + BaP, the digestive gland showed low stress.

## 4. Discussion

Microplastics are a major concern to marine environment due to their bioavailability to organisms. Ingestion is the most important method by which many marine fauna interact with MPs, especially when the feeding mechanisms of the organisms are non-discriminatory and do not allow them to differentiate between food and plastic fragments [39,40]). The results did not demonstrate significant differences of ingestion between different MPs sizes (Figure 1). In a similar study, however, the ingestion of MPs in clams exposed to 20–25 μm uncontaminated LDPE MPs was significantly higher than in clams exposed to smaller 4–6 μm MPs [41]. On the other hand, the accumulation of BaP in clams’ whole soft tissues showed that BaP accumulation of clams exposed to 4–6 μm MPs + BaP was lower than in clams exposed to bigger MPs (20–25 μm) (Table 2), indicating that BaP accumulation increased with size and larger particles deliver BaP better to the organisms. Moreover, it seems that smaller MPs (4–6 μm are rapidly excreted in bulk, while larger ones are excreted more slowly, having more time to be retained for BaP desorption according to Kinjo et al. [42]. This is in line with findings by Ribeiro et al. [8] that exposed *S. plana* to 20 µm PS (polystyrene) MPs and noted that MPs were still retained after a week of depuration. In long-term exposure of *Mytilus galloprovincialis* to two different sizes of PS MPs (0.5–4.5 μm), BaP accumulation also increased with a size increase in PS MPs, while the ingestion of MPs decreased with increasing size [43].

Gills tissues, which are the major organ responsible for filtration, are the first site of MPs uptake in mussels [6,8], with *S. plana* also ingesting particles through inhalation siphon, being consequently transported to the mouth and digestive gland [20]. The present results indicate a time and tissue-dependent oxidative stress response that varies depending on the treatment used and the biomarker assessed. SOD activity showed a bell shape behavior in gills tissues exposed to 20–25 µm MPs + BaP with the highest level being reached after 7 days of exposure (Figure 2A). The increase in SOD activity indicates that the first line of defense is protecting the cell [44,45] against oxidative stress produced as a result of clam’s exposure to MPs with BaP adsorbed. O’Donovan and colleagues [9] also observed a similar behaviour in clams exposed to 10–13 µm MPs with adsorbed BaP hypothesizing that the observed increase could result from the toxicity of the adsorbed contaminant. A similar behavior was also noted by Ribeiro et al. [8] showing an increase in SOD levels in the same clam species exposed to the same size (20 µm) but to polystyrene MPs. Taking into consideration that BaP accumulation was higher in clams exposed to 20–25 µm (Table 2) and SOD activity was also higher in the gills of clams exposed to the bigger particles, the likely higher retention time for larger MPs particles is apparently influencing BaP accumulation effects in this species. However, in all MPs exposed clams, a decrease in SOD activity was detected after 14 days, indicating that SOD was unable for balancing oxidative stress induced by prolonged exposure to MPs. In the digestive gland, however, SOD activity had little variation between treatments potentially indicating that if there was an increase in ROS production in this tissue, SOD was not acting as a mechanism of defense.

Catalase is the second line of defense, preventing cellular damage caused by ROS by reducing H_2_O_2_ to H_2_O and O_2_ [46,47]. ROS are reactive chemical species containing oxygen that play an important role in apoptosis induction under both physiologic and pathologic conditions [48,49]. In the gills, CAT activity remained unchanged in clams exposed to virgin MPs with exposure time (Figure 2C) being much lower than in the digestive gland (Figure 2D). This response indicates that CAT activity is unlikely for the antioxidant mechanism used by *S. plana* when exposed to virgin MPs, or the exposure time was not enough to induce significant responses. However, in clams exposed to both sizes of MPs with BaP adsorbed, CAT activity increased and was not able to cope with ROS, which was also supported by PCA analysis (Figure 3). An inhibition of CAT activity in the digestive gland of the marine mussel *M. galloprovincialis* was also reported by Avio et al. [5] after exposure to 1000–100 µm and <100 µm MPs (PE and PS). Ribeiro et al. [8], shows an increase in CAT activity in *S. plana* gills after three days of exposure to PS MPs (20 µm) while in the digestive gland CAT was inhibited after 7 days of exposure. Similarly, Gonzalez-Soto et al. [41] also detected an increase in CAT activity in the mussels *M. galloprovincialis* exposed to PS MPs contaminated with BaP of two different sizes. BaP is known to induce oxidative stress in mussels *M. galloprovincialis* [50]. In the digestive gland, IBR for MPs without BaP adsorbed was higher (Table 3), which might result from the responses of antioxidant enzymes (SOD and CAT) given the apparent greater negative effects MPs in digestive gland. This is reflected in the HIS index that indicate a lower health status for the digestive gland, where the up taken particles remained longer for digestion (Table 3).

GST is not only a phase II biotransformation enzyme involved in detoxification metabolism but also has an antioxidant role in the metabolism of lipophilic organic compounds. In the latter, it catalyzes the conjugation of the reduced form of glutathione (GSH) to xenobiotic substrates and is involved in detoxification by converting reactive lipophilic molecules into non-reactive ones facilitating their excretion by the organism [45,51,52,53]. BaP is highly lipophilic and *S. plana* uses this detoxification mechanism to deal with the exposure of BaP adsorbed to MPs in gills, given the observed significant increase in GST activity for 20–25 µm MPs + BaP after 7 days (*p* < 0.05) (Figure 2E). GST activity was not induced in the digestive gland throughout the time of exposure (Figure 2F), which was also confirmed by PCA analysis (Figure 3). Ribeiro et al. [8] also showed GST activity increase in the gills of *S. plana* when exposed to 20 µm PS MPs. A short response of GST activity seems to be triggered, likely in response to ROS formation, but only for the BaP contaminated larger size particles.

Oxidative damage happens when oxidative stress reaches a certain level that may cause damage to DNA, proteins carbohydrates and lipid membranes. Lipid peroxidation is the process in which free radicals react in cell membranes to form lipid hydroperoxides resulting in cell damage [46]. LPO levels were treatment and tissue dependent, showing a significant increase in LPO levels in the gills of clams exposed to LDPE + BaP MPs (4–6 µm) and in the digestive gland exposed to both virgin and contaminated smaller LDPE MPs (4–6 µm) after 7 days (Figure 2H,I). In this manner, it may be hypothesized that an increase in LPO may result from inefficient oxidative stress response mechanism in processing the excess of ROS when exposed to smaller particles. This is reflected in IBR, calculated for clam gills and digestive gland indicating that in gills the smaller size range of MPs has a higher IBR level than bigger MPs, hence greater negative ecotoxicological effects to the clam’s gills (Table 3).

Acetylcholinesterase is responsible for the break down of acetylcholine and other choline esters responsible for neurotransmission found in neuromuscular junctions and chemical synapses of the cholinergic type [17,18]. AChE activity increased over time in all treatments but was only significant for 4–6 μm and 20–25 μm MPs +BaP, showing no inhibition for AChE activity (Figure 2G). In organisms exposed to MPs, an inhibition of AChE activity would be expected as observed in juveniles of the common goby *Pomatoschistus microps* exposed to PE microspheres (1–5 μm) for 96 h, with and without absorbed contaminants (pyrene) [44]. Ribeiro et al. [8] also described an inhibition response of AChE activity to PS MPs exposure of *S. plana*. However, O’Donovan et al. [9] also showed an increase in AChE activity in the same clam species after the same time of exposure to LDPE MPs (11–13 μm) contaminated with the same concentration of BaP, indicating that there is an induction of AChE as a result of MPs exposure. This might indicate that neurotoxic effects could be dependent of the chemical composition of MPs.

## 5. Conclusions

All MP sizes were ingested in whole clam tissues. The higher accumulation of BaP occurred when clams were exposed to larger MPs. Antioxidant enzyme SOD has a higher response in gills for the larger size of LDPE + BaP MPs and an increase in GST activity in the gills of the clams exposed to 20–25 μm MPs + BaP after 7 days of exposure was observed. AChE had no inhibition response for any MP treatments. LPO levels were higher in the digestive gland of clams exposed to smaller virgin size LDPE MPs (4–6 μm) with and without BaP. IBR indicated greater negative effects in the gills and in LDPE MPs of smaller size (4–6 μm). HIS demonstrates that the digestive gland was more affected and that the smaller size of MPs (4–6 μm) produced the worst effects in both tissues irrespective of being with or without BaP adsorbed. It can be concluded that MP effects are size-dependent and that MPs can be a vector of BaP exposure but further studies analyzing other effects might be needed to clarify the biological effects of the different sizes of LPDE MPs with and without BaP adsorbed.

## Figures and Tables

**Table 1:** Condition index (mean± SD) from each treatment (control, 4–6 µm MPs, 4–6 µm MPs+ BaP, 20–25 MPs µm and 20–25 µm MPs + BaP) at different times of exposure.

Treatment/Time (days)	0	7	14
**Control**	12.1 ± 0.4	10.8 ± 0.4	8.0 ± 0.5
**4–6 µm MPs**	12.1 ± 0.4	8.8 ± 0.4	12.0 ± 0.4
**4–6 µm MPs + BaP**	12.1 ± 0.4	8.7 ± 0.7	6.6 ± 0.4
**20–25 µm**	12.1 ± 0.4	12.0 ± 0.4	9.5 ± 0.5
**20–25 µm MPs + BaP**	12.1 ± 0.4	9.0 ± 0.4	8.7 ± 0.2

**Table 2:** Benzo-a pyrene concentration (µg/kg w.w.) after 14 days of exposure.

Treatment	Benzo-a Pyrene (μg/kg w.w.)
Control (0)	1.00
Control (14)	0.50
4–6 μm	0.61
4–6 μm MPs + BaP	2.67
20–25 μm	0.67
20–25 μm MPs + BaP	4.94

**Table 3:** IBR and HIS in the gills and digestive glands for all treatments. A (Healthy); B (Low Stress); C (Medium Stress); E (Pathological).

Index	Treatment	Gills	Digestive Gland
**IBR**	4–6 µm MPs	8.5	6
	4–6 µm MPs + BaP	9	4.5
	20–25 µm	7	5
	20–25 µm MPs + BaP	7.5	4
**HIS**	4–6 µm MPs	A+	E
	4–6 µm MPs + BaP	E	C
	20–25 µm	A+	C+
	20–25 µm MPs + BaP	A	B

## Data Availability

Data available in a publicly accessible repository that does not issue DOIs.

## References

[1] Barnes D. (2005). Remote Islands Reveal Rapid Rise of Southern Hemisphere Sea Debris. Sci. World J..

[2] Henderson J. (2001). A Pre- and Post-MARPOL Annex V Summary of Hawaiian Monk Seal Entanglements and Marine Debris Accumulation in the Northwestern Hawaiian Islands, 1982–1998. Mar. Pollut. Bull..

[3] Derraik J.G.B. (2002). The pollution of the marine environment by plastic debris: A review. Mar. Pollut. Bull..

[4] Thompson R.C., Olsen Y., Mitchell R.P., Davis A., Rowland S.J., John A.W.G., McGonigle D., Russell A.E. (2004). Lost at sea: Where is all the plastic?. Science.

[5] Avio C.G., Gorbi S., Milan M., Benedetti M., Fattorini D., d’Errico G., Pauletto M., Bargelloni L., Regoli F. (2015). Pollutants bioavailability and toxicological risk from microplastics to marine mussels. Environ. Pollut..

[6] Browne M.A., Dissanayake A., Galloway T.S., Lowe D.M., Thompson R.C. (2008). Ingested microscopic plastic translocates to the circulatory system of the mussel, *Mytilus edulis* (L.). Environ. Sci. Technol..

[7] Cole M., Lindeque P., Fileman E., Halsband C., Goodhead R., Moger J., Galloway T.S. (2013). Microplastic ingestion by zooplankton. Environ. Sci. Technol..

[8] Ribeiro F., Garcia A.R., Pereira B.P., Fonseca M., Mestre N.C., Fonseca T.G., Bebianno M.J. (2017). Microplastics effects in *Scrobicularia plana*. Mar. Pollut. Bull..

[9] O’Donovan S., Mestre N.C., Abel S., Fonseca T.G., Carteny C.C., Cormier B., Keiter S.H., Bebianno M.J. (2018). Ecotoxicological Effects of Chemical Contaminants Adsorbed to Microplastics in the Clam *Scrobicularia plana*. Front. Mar. Sci..

[10] Boerger C.M., Lattin G.L., Moore S.L., Moore C.J. (2010). Plastic ingestion by planktivorous fishes in the North Pacific central gyre. Mar. Pollut. Bull..

[11] Carbery M., O’Connor W., Thavamani P. (2018). Trophic transfer of microplastics and mixed contaminants in the marine food web and implications for human health. Environ. Inter..

[12] Caruso G. (2019). Microplastics as vectors of contaminants. Mar. Pollut. Bull..

[13] Cousin X., Batel A., Bringer A., Hess S., Begout M.-L., Braunbeck T. (2020). Microplastics and sorbed contaminants-Trophic exposure in fish sensitive early life stages. Mar. Environ. Res..

[14] Teuten E.L., Saquing J.M., Knappe D.R.U., Barlaz M.A., Jonsson S., BjÃrn A., Rowland S.J., Thompson R.C., Galloway T.S., Yamashita R. (2009). Transport and release of chemicals from plastics to the environment and to wildlife. Philos. Trans. R. Soc. B Biol. Sci..

[15] Batel A., Borchert F., Reinwald H., Erdinger L., Braunbeck T. (2018). Microplastic accumulation patterns and transfer of benzo[a]pyrene to adult zebrafish (*Danio rerio*) gills and zebrafish embryos. Environ. Pollut..

[16] Liu T., Pan L., Jin Q., Cai Y. (2015). Differential gene expression analysis of benzo (a) pyrene toxicity in the clam. Ruditapes philippinarum. Ecotoxicol. Environ. Saf..

[17] Pogribny I.P., Hainaut P., Boffetta P. (2019). Environmental Exposures and Epigenetic Perturbations. Encyclopedia of Cancer.

[18] Viarengo A., Lowe D., Bolognesi C., Fabbri E., Koehler A. (2007). The use of biomarkers in biomonitoring: A 2-tier approach assessing the level of pollutant-induced stress syndrome in sentinel organisms. Comp. Biochem. Physiol. Part C Toxicol. Pharmacol..

[19] Tsangaris C., Kormas K., Strogyloudi E., Hatzianestis I., Neofitou C., Andral B., Galgani F. (2010). Multiple biomarkers of pollution effects in caged mussels on the Greek coastline. Comp. Biochem. Phys. C.

[20] Hughes R. (1969). A study of feeding in Scrobicularia plana. J. Mar. Biol. Assoc. U. K..

[21] Dixon D. (2002). Marine invertebrate eco-genotoxicology: A methodological overview. Mutagenesis.

[22] Beliaeff B., Burgeot T. (2002). Integrated biomarker response: A useful tool for ecological risk assessment. Environ. Toxicol. Chem..

[23] Serafim A., Company R., Lopes B., Fonseca V.F., França S., Vasconcelos R.P., Bebianno M.J., Cabral H.N. (2012). Application of an integrated biomarker response index (IBR) to assess temporal variation of environmental quality in two portuguese aquatic systems. Ecol. Indic..

[24] Dagnino A., Allen J.I., Moore M.N., Broeg K., Canesi L., Viarengo A. (2007). Development of an expert system for the integration of biomarker responses in mussels into an animal health índex. Biomarkers.

[25] Larsson M., Hagberg J., Rotander A., Van Bavel B., Engwall M. (2013). Chemical and bioanalytical characterisation of PAHs in risk assessment of remediated PAH-contaminated soils. Environ. Sci. Pollut. Res..

[26] Maranho L., Baena-Nogueras R., Lara-Martín P., DelValls T., Martín-Díaz M. (2014). Bioavailability, oxidative stress, neurotoxicity and genotoxicity of pharmaceuticals bound to marine sediments. The use of the polychaete *Hediste diversicolor* as bioindicator species. Environmental Research. Environ. Res..

[27] Walne P. (1976). Experiments on the culture in the sea of the butterfish *Venerupis decussata* L.. Aquaculture.

[28] De Witte B., Devriese L., Bekaert K., Hoffman S., Vandermeersch G., Cooreman K., Robbens J. (2014). Quality assessment of the blue mussel (Mytilus edulis): Comparison between commercial and wild types. Mar. Pollut. Bull..

[29] McCord J.M., Fridovich I. (1969). Superoxide dismutase an enzymic function for erythrocuprein (hemocuprein). J. Biol. Chem..

[30] Greenwald R.A. (1987). Handbook of methods for oxygen radical research. Free Radic. Biol. Med..

[31] Habig W.H., Pabst M.J., Jakoby W.B. (1974). Glutathione S-transferases the first enzymatic step in mercapturic acid formation. J. Biol. Chem..

[32] McFarland V., Inouye L., Lutz C., Jarvis A., Clarke J., McCant D. (1999). Biomarkers of Oxidative Stress and Genotoxicity in Livers of Field-Collected Brown Bullhead, *Ameiurus nebulosus*. Arch. Environ. Contam. Toxicol..

[33] Hayes J.D., Flanagan J.U., Jowsey I.R. (2005). Glutathione Transferases. Annu. Rev. Pharmacol. Toxicol..

[34] Ellman G., Courtney K., Andres V., Featherstone R. (1961). A new and rapid colorimetric determination of acetylcholinesterase activity. Biochem. Pharmacol..

[35] Colovic M., Krstic D., Lazarevic-Pasti T., Bondzic A., Vasic V. (2013). Acetylcholinesterase Inhibitors: Pharmacology and Toxicology. Curr. Neuropharmacol..

[36] Erdelmeier I., Gérard-Monnier D., Yadan J., Chaudière J. (1998). Reactions of N—Methyl-2-phenylindole with Malondialdehyde and 1998, 4-Hydroxyalkenals. Mechanistic Aspects of the Colorimetric Assay of Lipid Peroxidation. Chem. Res. Toxicol..

[37] Bradford M. (1976). A Rapid and Sensitive Method for the Quantitation of Microgram Quantities of Protein Utilizing the Principle of Protein-Dye Binding. Anal. Biochem..

[38] Sanchez W., Burgeot T., Porcher J.-M. (2013). A novel “Integrated Biomarker Response” calculation based on reference deviation concept. Environ. Sci. Pollution. Res..

[39] Lusher A. (2015). Microplastics in the marine environment: Distribution, interactions and effects. Marine Anthropogenic Litter.

[40] Allen A.S., Seymour A.C., Rittschof D. (2017). Chemoreception drives plastic consumption in a hard coral. Mar. Pollut. Bull..

[41] Islam N., Garcia da Fonseca T., Vilke J., Gonçalves J.M., Pedro P., Keiter S., Cunha S.C., Fernandes J.O., Bebianno M.J. (2021). Perfluorooctane sulfonic acid (PFOS) adsorbed to polyethylene microplastics: Accumulation and ecotoxicological effects in the clam *Scrobicularia plana*. Mar. Environ. Res..

[42] Kinjo A., Mizukawa K., Takada H., Inoue K. (2019). Size-dependent elimination of ingested microplastics in the Mediterranean mussel *Mytilus galloprovincialis*. Mar. Pollut. Bull..

[43] González-Soto N., Hatfield J., Katsumiti A., Duroudier N., Lacave J.M., Bilbao E., Orbea A., Navarro E., Cajaraville M.P. (2019). Impacts of dietary exposure to different sized polystyrene microplastics alone and with sorbed benzo[a]pyrene on biomarkers and whole organism responses in mussels *Mytilus galloprovincialis*. Sci. Total Environ..

[44] Livingstone D.R. (2001). Contaminant-stimulated reactive oxygen species production and oxidative damage in aquatic organisms. Mar. Pollut. Bull..

[45] Van der Oost R., Porte-Visa C., van den Brink N. (2005). Ecotoxicological Testing of Marine and Freshwater Ecosystems.

[46] Oliveira M., Maria V., Ahmad I., Serafim A., Bebianno M., Pacheco M., Santos M. (2009). Contamination assessment of a coastal lagoon (Ria de Aveiro, Portugal) using defence and damage biochemical indicators in gill of *Liza aurata*—An integrated biomarker approach. Environ. Pollut..

[47] Solé M., Kopecka-Pilarczyk J., Blasco J. (2009). Pollution biomarkers in two estuarine invertebrates, *Nereis diversicolor* and *Scrobicularia plana*, from a Marsh ecosystem in SW Spain. Environ. Int..

[48] Simon H.U., Haj-Yehia A., Levi-Schaffer F. (2000). Role of reactive oxygen species (ROS) in apoptosis induction. Apoptosis.

[49] Pittura L., Avio C.G., Giuliani M.E., d’Errico G., Keiter S.H., Cormier B., Regoli F. (2018). Microplastics as vehicles of environmental PAHs to marine organisms: Combined chemical and physical hazards to the Mediterranean mussels, *Mytilus galloprovincialis*. Front. Mar. Sci..

[50] Maria V.L., Bebianno M.J. (2011). Antioxidant and lipid peroxidation responses in *Mytilus galloprovincialis* exposed to mixtures of benzo(a)pyrene and copper. Comp. Biochem. Physiol. C Toxicol. Pharm..

[51] Hoarau P., Garello G., Gnassia-Barelli M., Romeo M., Girard J. (2002). Purification and partial characterization of seven glutathione S -transferase isoforms from the clam *Ruditapes decussatus*. Eur. J. Biochem..

[52] Lesser M.P. (2006). Oxidative stress in marine environments: Biochemistry and physio- logical ecology. Annu. Rev. Physiol..

[53] Nikinmaa M. (2014). An Introduction to Aquatic Toxicology.

